# Comparing the Effect of Thermal-Oxidation and Photo-Oxidation of Asphalt Mixtures on the Rheological and Chemical Properties of Extracted Bituminous Binder

**DOI:** 10.3390/ma15196793

**Published:** 2022-09-30

**Authors:** Ahmed Abouelsaad, Greg White

**Affiliations:** School of Science, Technology and Engineering, University of the Sunshine Coast, Sippy Downs, QLD 4556, Australia

**Keywords:** asphalt, aging, accelerated, laboratory, thermal oxidation, photo-oxidation

## Abstract

The reliable and representative ageing of asphalt samples in the laboratory is critically important to research on asphalt durability, waste material recycling and rejuvenation treatments. However, standard laboratory ageing protocols omit ultraviolet radiation and moisture, and are also based on a universally applied oven temperature and ageing duration. The aim of this research was to demonstrate the importance of ultraviolet radiation in laboratory asphalt aging, motivated by the need for more realistic ageing protocols. Asphalt cores were thermally aged in a standard laboratory oven for 98 days, while other cores were aged for the same period in a weathering chamber that combined thermal–oxidative (heat) and photo-oxidative (ultraviolet irradiation) aging. The bitumen was then extracted from the top, middle and bottom of each asphalt core, and tested for rheological, chemical, and compositional properties. The results were used to compare the effects of the aging protocols, and the effects of depth below the pavement surface. It was concluded that accelerated laboratory asphalt ageing must include photo-oxidation, in combination with thermal oxidation. It was also concluded that both chemical and rheological properties were effective indicators of extracted asphalt binder aging, although the rheological testing was preferred.

## 1. Introduction

A pavement designer generally has the main objective of controlling load-related distress, including subgrade rutting and asphalt fatigue. However, the surface damage caused by exposure to environmental factors leads to periodic surface replacement, even in the absence of fatigue and rutting distress [1]. Exposure to the environment results in bitumen aging, which is defined as changes in the rheological or chemical properties and composition of the binder with time [2]. Furthermore, bitumen aging causes changes in the overall properties of asphalt mixtures. Consequently, the mechanics of aging and accelerated laboratory aging of asphalt mixture samples has gained the interest of many pavement researchers in recent years [2].

Bitumen aging is comprised of two aspects; thermal–oxidative aging, which is mainly caused by heat in the existence of atmospheric oxygen [3,4,5], and photo-oxidative aging, caused by ultraviolet (UV) irradiation and oxygen [6]. Both heat and UV expose degrade the bituminous binder, but their effects are not the same [7]. Furthermore, a complex aging behaviour is noticed when both heat and UV irradiation are combined [8].

Current asphalt aging practice is based on thermal–oxidative aging and there are standard laboratory simulation protocols, including the Rolling Thin Film Oven (RTFO) [9] and Pressure Aging Vessel (PAV) [10] treatment of bituminous binder samples. The RTFO is intended to simulate aging during the production and placement of asphalt mixtures, while the PAV is intended to simulate long-term (7–10 years) aging in the field. In contrast, and despite its recognised importance, UV aging is not including in the standard protocols [11], which is expected to reflect the convenience and accessibility to laboratory ovens that do not have UV capability.

Some researchers believe that neither RTFO nor PAV is sufficient to simulate field aging of bituminous binders. First, current standards do not take into consideration some of the important environmental factors, including UV irradiation [12,13]. Second, applying the same protocols universally to all climatic region is unrepresentative and inappropriate [2]. Third, the effect of field aging is more severe on the top layer of asphalt, compared to deeper layers [14,15,16], which is known as the aging profile, but is not represented in thermal–oxidative standards. Furthermore, some field-aging studies have found that RTFO and PAV are insufficient to estimate 7–10 years of field aging [17]. Thus, developing a more realistic accelerated laboratory aging has gained the interest of researchers in recent times.

In recent times, several studies have been conducted on the effect of UV irradiation on the rheological properties of bitumen. Menapace et al. [13] found that combining UV irradiation and heat caused more noticeable chemical changes in the bitumen, compared to the standard RTFO/PAV protocols. Additionally, the samples aged in the UV weathering chamber showed significant differences in the chemical composition of the surface and deeper layers, which confirms the aging gradient with depth. Kuang et al. [18] also confirmed this finding and concluded that the combined effect of thermal and UV aging is different to the effect of heat only. Many other studies have also demonstrated the significant effect of UV irradiation on the rheological properties of bitumen [19,20,21,22].

Regarding the aging of compacted asphalt mixtures, AASHTO R30 [23] is the only standard aging protocol, in which compacted mixture samples are conditioned at 85 °C for 5 days. This is intended to reflect field exposure of somewhere between 5 years and 10 years, as estimated by the Strategic Highway Research Program (SHRP) based on limited field data [24]. However, this single time-temperature combination is not universally appropriate for all climatic conditions. Moreover, the conditioning temperature is still debatable, as it was found that 95 °C appears to be the most reasonable conditioning temperature of loose asphalt mixtures without altering oxidation reactions, as suggested by the NCHRP 09-54 project. In addition, even at 95 °C conditioning, 21 days of aging was found to approximate the level of oxidation of the binder extracted from the surface of 8 years field cores, in terms of chemical and rheological changes [24], while at 70 °C and 85 °C, 117 and 41 days were required, respectively.

Although many researchers have studied the distinct effects of combining UV irradiation and heat on the bituminous binders, fewer efforts have been directed to study this combination of effects on compacted asphalt mixture samples. It is expected that this reflects the additional effort required to extract the bituminous binder from the aged mixture samples, as well as the convenience of aging and testing binder samples. However, this convenience is not justified.

Grilli et al. [25] concluded that the degree of long-term aging is governed by many factors, including the binder film thickness around the aggregate, and the voids content of the mixture, because these parameters affect how the oxygen interacts with the bitumen in the asphalt sample. Therefore, understanding the changes in mechanical properties of mixture, and the chemical and rheological changes in the extracted binder, has gained the interest of researchers recently.

Abouelsaad and White [26] studied the effect of heat only, compared to the combined effect of heat and UV irradiation on asphalt mixture samples. It was concluded that the combined effect resulted in more severe aging, measured in terms of asphalt mixture resilient modulus and surface macro-texture. Furthermore, Yu et al. [27] used an accelerated weathering machine to simulate the actual field conditions including heat, UV and moisture and evaluated the changes in the extracted binder using Atomic Force Microscopy (AFM) and DSR response. It was concluded that 2400 h of weathering had the same effect as the combination of RTFOT and PAV, in terms of morphology, adhesion and modulus of elasticity of the samples.

Xiao et al. [28] compared the effect of aging using the traditional AASHTO R30 mixture protocol, and UV aging, by developing a custom-built oven with UV lamps. The results showed that fracture energy of unaged samples was higher, compared to samples aged using both the R30 and UV protocols, and both aging protocols were associated with similar fracture energy values, although the results were slightly higher for the R30 protocol aged samples. Furthermore, Hagos [29] combined oxidative aging using heat, along with UV radiation in a weathering chamber to simulate field aging of asphalt mixtures. Different combinations of heat, rain, humidity and UV radiation were applied for 185 h. It was concluded that the new and modified laboratory techniques could not represent the adverse effects observed for even one year of field aging, which certainly warrants further study with longer exposure times, and additional changes to the combinations of aging factors and the accelerated aging duration.

Regardless of the aging protocol used, it is clear that aging affects the chemical and rheological properties of the bituminous binder and most researchers have characterized the degree of aging based on rheological and chemical testing, including the response to Dynamic Shear Rheometer (DSR), Fourier-transform infrared spectroscopy (FT-IR) testing and more recently, the relative portions of the Saturates, Aromatics, Resins and Asphaltenes (SARA) fractions.

DSR determined complex shear modulus of unaged and aged samples is an established technique to characterize binder aging, as suggested by many researchers [30,31,32]. In terms of the chemical properties, FT-IR spectroscopy provides the infrared spectrum of transmission or absorption of a fuel sample, and has been widely used as an indicator of the aging behaviour of bitumen [33,34]. From another perspective, measuring the polarity-based distribution of what are known as the SARA fractions can also be used as an indicator of chemical changes that occur as a result of the bitumen aging [35]. All these approaches have been used by various researchers are all are potential indicators of asphalt mixture aging.

Towards a more realistic aging procedure, and due to the significant shortcomings of using a single temperature-time conditioning, in addition to the limited number of studies into the effect of UV irradiation on the properties of bituminous binder extracted from aged asphalt mixture samples, this study has the main objective of determining the different effects of heat only (thermal oxidation), compared to a combination of heat and UV radiation (photo-oxidation). The chemical and rheological properties of bitumen extracted from asphalt cores, before and after ageing. In addition, the effect of depth on the chemical and rheological properties of the extracted bitumen, after thermal–oxidative aging and photo-oxidative aging, was also considered.

## 2. Materials and Methods

### 2.1. Materials

Six dense-graded asphalt (DGA) cores were recovered from the newly constructed runway surface of Emerald airport, which is located near the town of Emerald in Queensland, Australia [36]. The recovered cores were aged in different weathering environments. Three cores were conditioned in a conventional oven at 70 °C, and three cores were conditioned at the same temperature, with the addition of UV irradiation in a commercial weathering chamber. The binder was extracted from the top, middle and bottom of the aged and unaged core samples, before being tested in the DSR, by the FT-IR and for SARA analysis.

The asphalt mixture was a nominal 14 mm-sized DGA for runway surfacing, with volumetric compositions meeting the requirements of the Australian airport asphalt specification, as detailed in Table 1, and A10E polymer modified bituminous binder was used, with properties summarised in Table 2. The A10E is highly modified with styrene-butadiene-styrene (SBS) polymer and is commonly used for airport pavement surfacing and other heavy duty asphalt production in Australia [37]. Recovered cores had a nominal diameter of 143 mm and an average thickness of 44 mm, after cutting the bottom of the cores to remove the irregular edges.

### 2.2. Experimental Work

#### 2.2.1. Aging Procedures

As stated above, the main purpose of this research was to compare thermal–oxidative aging and photo-oxidative aging effects on the rheological and chemical properties of extracted bitumen from artificially aged DGA cores. A commercially available Suntest XXL weathering chamber from Atlas Material Testing Solutions was used to simulate the photo-oxidative aging. Unlike other weathering chambers, the Suntest XXL uses xenon lamps to simulate sunlight according to the CIE85 reference sunlight, with irradiance range 30–65 w/m^2^ and control of irradiance either from 300 nm to 400 nm, or 340 nm as a single representative wavelength. The advantage of using xenon lamps over traditional fluorescent lamps is a more realistic simulation of sunlight over a wide range of wavelengths. In contrast, traditional fluorescent lamps are effective at simulating the short-wave UV (<365 nm) portion of the spectrum, but not the longer wavelengths [38]. Li et al. [39] studied the effect of UV wavelength on asphalt aging and concluded that under the same aging condition, each UV wavelength has an important and different effect on the chemical and physical property changes of the bitumen, meaning that a realistic irradiation spectrum is important for aging asphalt mixture samples. The weather chamber settings are summarized in Table 3.

#### 2.2.2. Cutting Samples and Extractions

**Figure 1:**
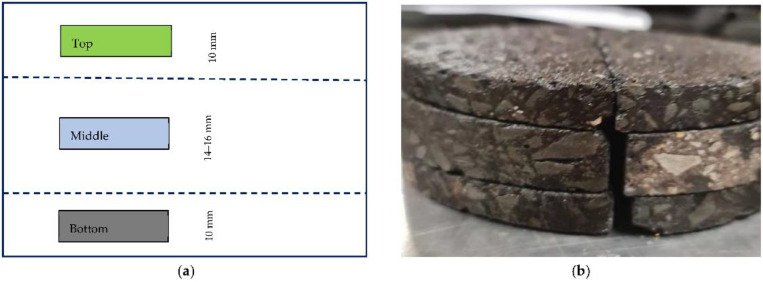
Core cutting plan (**a**) schematic and (**b**) example.

After aging, bituminous binder was extracted from each core. To obtain binder extractions representative of different depths below the pavement surface, the asphalt samples were first cut into three disks. Practically, it was impossible to control the thickness of all the disks, so a simplified cutting procedure was followed. First, each core was cut into two semi-circular cylinders. Next, each half-core was sliced into three half-disks; referred to as the top, middle and bottom. The top and bottom disks were cut to a nominal thickness of 10 mm, with the middle disk the remaining thickness, after losses due to sawing of the samples. The middle disks had a thickness of 14–16 mm, depending on the initial thickness of the core and the cutting losses. Figure 1 shows a schematic, and an example, of the cutting stages, as well as the six half-disks after cutting one core sample.

The bituminous binder was extracted from each half-disc and classified into categories; including top oven, middle oven, bottom oven, top UV, middle UV and bottom UV, according to the aging procedure used and the disk position. Three cores for each aging procedure were tested separately in order to confirm the repeatability of results, with a total number of 18 extracted bitumen samples. Binder extraction involved heating the asphalt disks to 160 °C, to break the disks into loose asphalt. After cooling, the loose asphalt was placed in a toluene bath for two hours to dissolve the binder. The dissolved asphalt mixture was then centrifuged for 15 min, and then the binder was separated from the solvent using a rotary evaporator.

#### 2.2.3. Extracted Bitumen Testing

To evaluate the effect of asphalt mixture aging on the extracted bitumen, three test methods were used:

- Response to DSR;
- FT-IR fingerprinting;
- SARA fraction analysis.

The rheological properties of the different bitumen samples were evaluated using a Kinexus DSR rheometer (Netzsch, Selb, Germany). A frequency sweep test was performed from 10 to 0.1 Hz. The testing temperature ranged from 30 °C to 70 °C. An 8 mm plate with a 2 mm gap was used for temperatures from 50 °C to 70 °C, while a 25 mm plate with a 1 mm gap was used for temperature from 30 °C to 50 °C.

A Thermo Nexus FT-IR spectrophotometer (Thermo Fisher Scientific, Waltham, WA, USA) equipped with Omnic 6.2 software was used to acquire the chemical spectra, also known as the bitumen fingerprint. Samples were scanned separately, and an average spectrum of each binder sample category was drawn to clarify the distinct effect of different aging procedures and disk position on the bitumen chemistry. The scanned ss ranged from 4000 cm^−1^ to 400 cm^−1^, and the aging effect was studied by following the changes of two characteristic infrared bands; the carbonyl band around 1700 cm^−1^, and the sulfoxide band around 1035 cm^−1^, as recommended in the literature [33,34].

SARA analysis was performed using an Iatroscan Thin Layer Chromatography with Flame Ionization Detection (TLC-FID) device (Iatroscan Europe, Bechenheim, Germany), following the established method for quantitating SARA fractions if bituminous binders. This provided an indication of the severity of the aging effect in terms of the relative chemical composition of the binder. In this method, the bitumen was separated into the four components, based on affinity to different solvents. In the first step, a small amount of bitumen (nominally 0.1 g) was dissolved into 25 mL of dichloromethane and then placed on chroma rods and treated with different solvents in development tanks. The development tanks contained hexane, toluene and dichloromethane/methanol. Hexane was used to separate the saturates, toluene was used to separate aromatics, dichloromethane/methanol was used to separate the resins, and the remaining sample was the asphaltenes. The soaking and drying time of each development tank is summarized in Table 4. Finally, the chroma rod rack was placed in the Iatroscan device (Iatroscan Europe, Bechenheim, Germany) to quantify each fraction.

## 3. Results and Discussion

### 3.1. Dynamic Shear Rheometer Results

**Figure 2:**
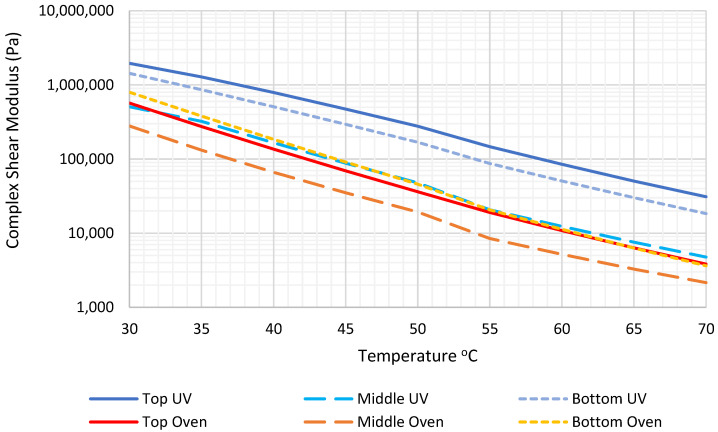
Temperature and average complex modulus at 1 Hz reference frequency.

The results from the DSR are represented as the average complex shear modulus versus temperature, at an average frequency of 1 Hz, as shown in Figure 2. The results clearly show the effect of UV combined with heat, as a more severe asphalt aging condition than the heat only applied in the conventional oven. The results also show that the top layer of each asphalt core was much more affected by UV and temperature, compared to the middle layer. However, the bottom layers showed higher complex modulus values than the middle layers. This reflects the thermal conductivity of the surface on which the cores were set, both in the oven or the UV chamber. The results of the oven samples also show that there was no significant difference between the top and bottom disks of the cores, with a slightly larger complex shear modulus of the bottom layer compared to the top layer, which can be explained in terms of the aggressive effect of heat, due to the high thermal conductivity of the oven base, compared to the thermal conductivity of the air surrounding the sides and tops of the samples. In the future, the bottom of the core samples should be thermally insulated from the base of the oven and the UV chamber.

**Figure 3:**
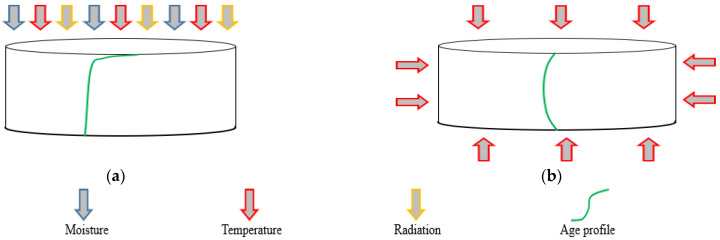
Schematic illustration of asphalt aged in the (**a**) field and (**b**) oven.

Studying the effect of aging at different depths from the pavement surface, known as the aging profile, was one of the main objectives of this research. White and Abouelsaad [40] hypothesized that the aging profile is different when samples are artificially aged in an oven, because the cores are affected by heat from all directions, compared to samples in the field, where aging is primarily from the surface top only. Figure 3 shows this hypothesis in terms of a schematic aging profile; in the field, conditions are more closely represented by the UV chambers than by the traditional oven aging protocols.

**Figure 4:**
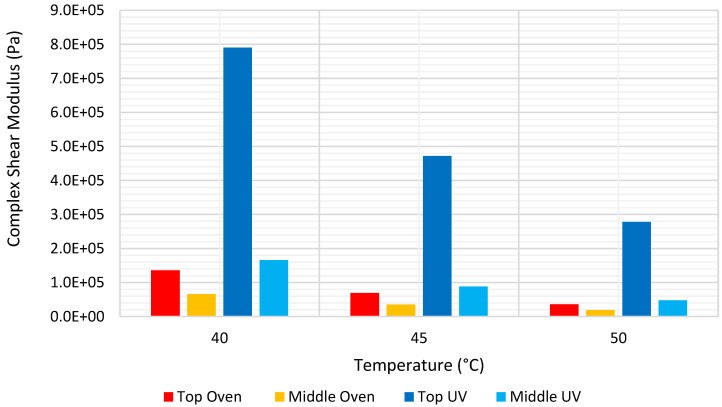
Complex modulus at 1 Hz reference frequency and mid-range temperatures.

To test this hypothesis, in terms of complex shear modulus, after discarding the bottom disks, which were compromised by the thermal conductivity of the sample platform, the top disk and middle disk results were compared. Figure 4 shows the complex shear modulus values at a frequency of 1 Hz, and at a temperature range of 40 °C to 50 °C, for the top and middle disks. The aging profile was observed for all samples, but this was more significant for the samples aged in the weather chamber. That is, the difference in complex modulus values for the top and middle layer was much greater for the samples aged in UV chamber, compared to the samples aged in the oven. Furthermore, Table 5 summarises the average complex shear moduli at different temperatures, as well as the ratio between the top and middle disks, which ranged from 1.8 to 2.1 for the samples aged in oven, while the equivalent ratio ranged from 3.9 to 7.1 for samples aged in the UV chamber. This supports the hypothesis of the limited effect of UV radiation to a small thickness of the pavement structure surface, which is important for accelerated aging of asphalt samples in the laboratory, but is not reflected by dry oven aging that is currently considered best practice. This also confirms White and Abouelsaad’s [40] hypothesis regarding the more significant aging profile associated with the weathering chamber.

### 3.2. SARA Analysis Results

SARA analysis is well established as an indicator of bituminous binder aging. However, because different bitumens have significantly different SARA compositions, focusing on the change in SARA fractions with age is important. Mirwald et al. [41] studied the effect of different aging techniques on three different binders, using the SARA fractions. It was concluded that all binders, regardless of the aging technique, showed similar changes in the four SARA fractions. A significant loss in aromatics was noticed with age, in parallel with an increase in the asphaltenes and resins contents, although the magnitudes of the changes was different. Furthermore, Zoroob and Airey [42] recommended the colloidal instability index (CI) as an indication of sol–gel behaviour of bitumen with age, as detailed in Equation (1). As the ratio increases, the gel behaviour of the bitumen becomes more pronounced, and the colloidal stability reduces. Laboratory tests confirm that the colloidal instability index increases with neat bitumen aging, but Kleiziene et al. [43] found that this is not the case for polymer modified binders (PMBs), where the index decreased with aging. It was concluded that in PMBs, polymer chain rupture occurs, which leads to a reduction in the reported asphaltene content, which causes a decrease in the index with PMB aging.

(1) $$\mathrm{CI}=\frac{\mathrm{Saturates}+\mathrm{Asphaltenes}}{\mathrm{Aromatics}+\mathrm{Resins}}$$

where CI = colloidal instability coefficient and Aromatics, Resins, Saturated and Asphaltenes are the portions of bitumen attributed to each fraction by the SARA analysis.

**Figure 5:**
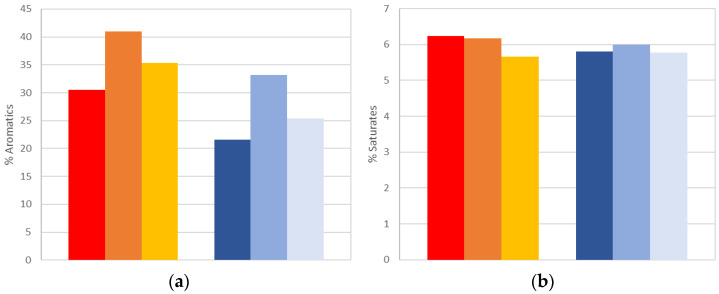

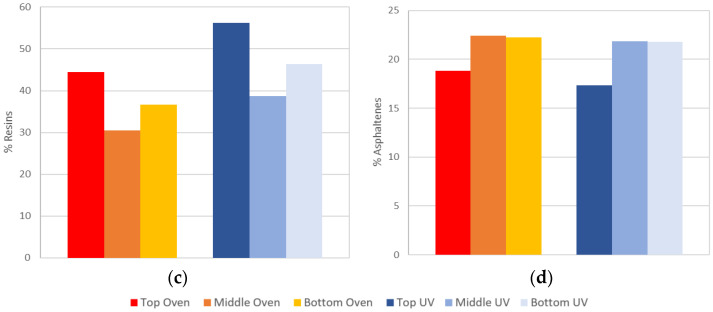
Comparison of SARA fraction values of extracted bitumen samples. (**a**) aromatics. (**b**) saturates. (**c**) resins. (**d**) asphaltenes.

**Figure 6:**
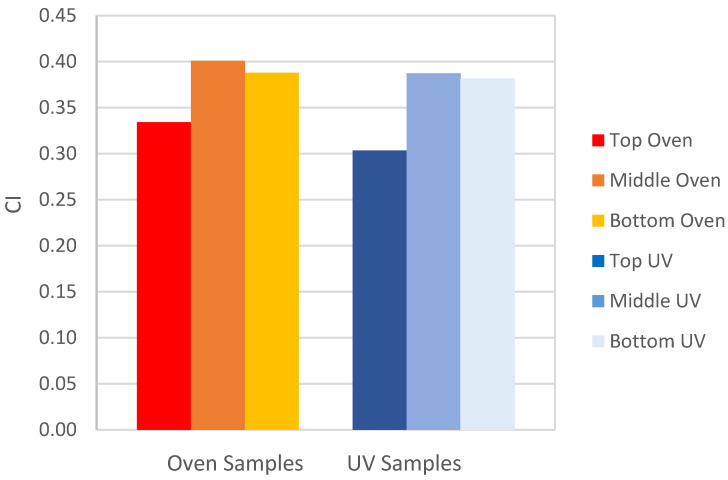
Average colloidal instability of extracted bitumen samples.

As previously explained, a SARA analysis of all extracted bitumen samples was performed. Table 6 and Table 7 summarize the results of the oven and UV aged samples, respectively. The average results of the four fractions are compared in Figure 5, while the calculated CI for each sample is in Figure 6.

A significant reduction in the aromatics and a comparable increase in the resins were noticed for the top disk of all artificially aged samples. Although this occurred in both the oven and the UV chamber aged samples, it was significantly greater for samples aged in the UV chamber. The asphaltene content reduced for the top disks, and this was comparable for the oven and UV chamber aged samples. In contrast, the saturates were generally unaffected by both the position in the asphalt core, and the aging treatment.

In terms of the colloidal instability index, as previously mentioned above, PMBs are associated with a decrease in index with aging [43]. Figure 6 reflects this with the top disks in both UV and oven aged samples associated with a lower colloidal instability index value, compared to the middle and bottom disks. This conflicts with unmodified bitumen aging, which is usually associated with an increase in CI. This phenomenon may be explained in terms of two possible scenarios. First, the polymer in the PMB may be reported as resin content during TLC-FID, due to polymer rupture during aging. Second, during aging, the polymer may combine with the resins to form longer chains, with higher molecular weights, making it harder to solute then in toluene and be captured by the chroma rod in the FID during the SARA analysis procedure. Further research is required to understand the different trend in CI for unmodified bitumens, compared to PMBs.

### 3.3. FT-IR Spectroscopy Results

**Figure 7:**
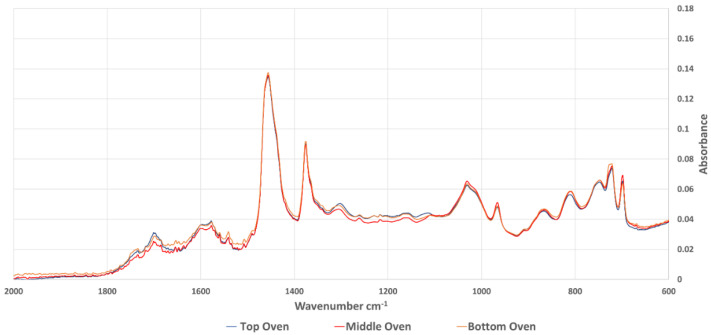
Whole FT-IR spectra for samples aged in the oven.

**Figure 8:**
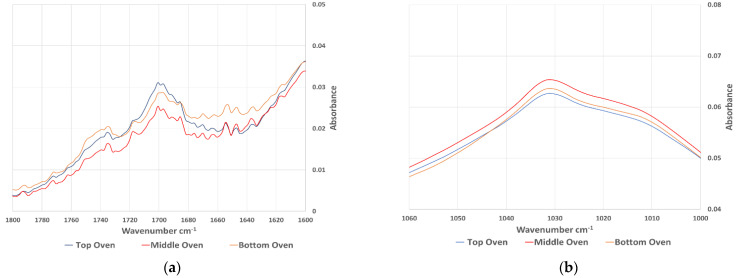
The (**a**) carbonyl and (**b**) sulfoxide peaks from FT-IR spectra of samples aged in the oven.

**Figure 9:**
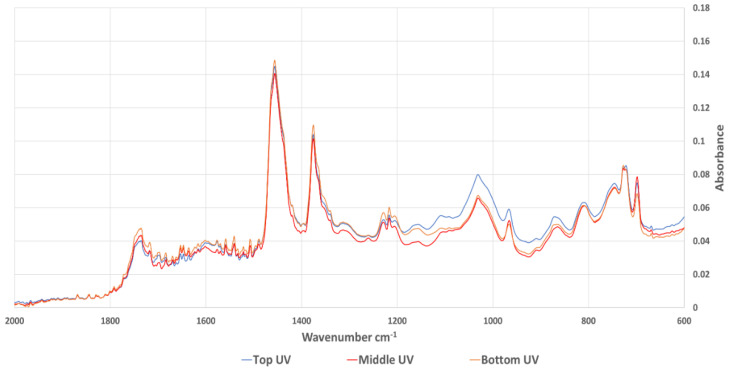
Whole FT-IR spectra for samples aged in the UV chamber.

**Figure 10:**
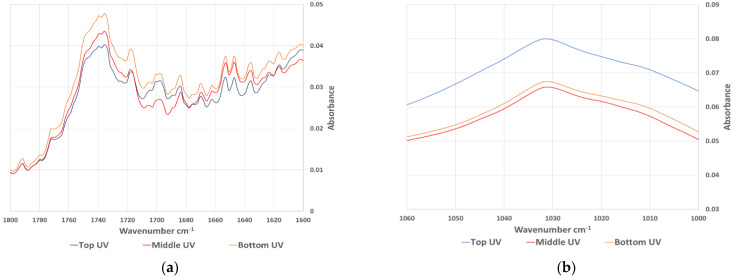
The (**a**) carbonyl and (**b**) sulfoxide peaks from FT-IR spectra of samples aged in the UV chamber.

FT-IR spectroscopy has been used in many studies to evaluate the aging behaviour of bitumen. Most of the studies noticed an increase in carbonyl (wavenumber 1660 cm^−1^–1800 cm^−1^) and sulfoxides (wavenumber 984 cm^−1^–1079 cm^−1^). Extracted bitumen samples were analysed using FT-IR and the average spectrum of each sample category was calculated as an average of absorbance at every wavenumber. Figure 7 shows the whole spectra of the samples aged in the oven, while Figure 8 shows the same results focused on the carbonyl and sulfoxide peaks. The equivalent spectra for the sample aged in the UV chamber are in Figure 9 (whole spectra) and Figure 10 (carbonyl and sulfoxide peaks).

To confirm the graphical representations, an aging index (AI) was calculated based on the carbonyl and sulfoxide peaks, as well as the reference aliphatic band (AI_ch3_) at the peak between wavenumber (1350 cm^−1^–1525 cm^−1^), according to Equation (2).

(2) $$\mathrm{AI}\left(\mathrm{FTIR}\right)=\frac{{\mathrm{AI}}_{\mathrm{CO}+}{\mathrm{AI}}_{\mathrm{SO}}}{{\mathrm{AI}}_{\mathrm{CH}3}}$$

where, AI(FTIR) is the aging index from the FTIR analysis, AI_CO_ = is the average of the sulfoxide peaks at 1030 cm^−1^, AI_SO_ = is the average of the carbonyl peaks at 1700 cm^−1^ and AI_CH3_ = the aliphatic band average peak between 1350 cm^−1^ and 1525 cm^−1^.

The calculated AI(FTIR) values are summarised in Table 8. This shows that the results of the FTIR analysis followed similar trends to those observed in the DSR and SARA results. The top disk of the samples aged in the UV chamber had a consistently higher AI compared to the top disk of samples aged in the oven, with an average ratio between the two aging protocols of 1.04. However, more significant differences were noticed between the top disk of UV aged sample, and the middle disk, with a ratio of 1.17, compared to oven samples, which had a ratio of just 1.02. This again demonstrates the importance of UV irradiation in accelerated asphalt mixture aging, particularly for creating the profile of aging that occurs during field aging.

## 4. Conclusions

Towards more realistic and reliable asphalt mixture aging in the laboratory, this research compared asphalt cores were aged in a conventional oven for 98 days, while cores were aged at the same temperature and for the same duration, but with the addition of UV irradiation. The bituminous binder was then extracted from the top, middle and bottom of each core sample and tested for rheology (DSR response), chemistry (FT-IT spectrum) and composition (SARA analysis).

The first objective of this study was to investigate the distinct effects of thermal–oxidative aging and photo-oxidative aging. It was concluded that the samples that were aged in a UV chamber showed a greater increase in complex shear modulus values, compared to samples that were aged in the conventional oven at the same temperature. This highlights the significance of UV irradiation on the contribution to rheological changes related to aging of asphalt mixtures. In addition, chemical changes determined by SARA analysis and FT-IR analysis, confirmed the rheological testing results. Average SARA analysis results of samples aged in UV chamber showed higher loss of aromatics, higher increase in resins content and lower CI values, all indications of more severe aging effects compared to samples aged in the oven. Moreover, FT-IR analysis followed the same trend as the DSR and SARA analysis, with slightly higher aging indices associated with the UV aged samples.

The second objective of this study was to understand the aging profile with depth below the asphalt surface. The ratios between the top 10 mm and the next 10 mm of asphalt were used as the indicator of the aging profile. For the DSR response, the aging profile was steeper for the samples aged with UV irradiation, compared to the samples aged in an oven, at the same temperature. Furthermore, for the FT-IR spectroscopy analysis, the average aging index ratio between the top and middle disks was similarly higher for the UV aged samples than those samples aged in the oven. Finally, for the colloidal stability from the SARA analysis, the ratio between the top and middle disks of samples aged in a UV chamber was lower than for the samples aged in the oven, which indicates the steeper aging profile associated with UV aging. That is, all three indicators of aging, being the rheology, chemistry and composition, indicated a significant aging profile, which was steeper for the samples exposed to UV radiation. That confirms the importance of UV irradiation of samples aged for research relating to pavement surfaces, which are exposed to UV irradiation in the field.

Overall, it was concluded that any future realistic aging acceleration procedure must include photo-oxidation by UV irradiation, in combination with thermal oxidation by heat. It was also concluded that the complex shear modulus measured by the DSR was the most efficient and clear indicator of relative aging of binder extracted from asphalt mixtures. However, the trends in the FTIR and SARA analyses were consistent with the rheological results, meaning that both chemical and rheological approaches to asphalt binder aging are effective.

## Figures and Tables

**Table 1:** Volumetric properties of DGA [36].

Property	Units	Test Method	Value
VMA	% (by volume)	AS/NZS 2891.8	14.4
VFB	% (by volume)	AS/NZS 2891.8	76
Air Voids	% (by volume)	AS/NZS 2891.8	4.2
Binder Content	% (by mass)	AS/NZS 2891.8	5.4
Binder Type	N/A	Selected by designer	A10E
Stability	kN	AS/NZS 2891.5	13.2
Flow	mm	AG:PT/T231	3.7
Tensile Strength	MPa	AG:PT/T232	1036
Resilient Modulus	MPa	AS/NZ 2891.13.1	1870
TSR	%	AG:PT/T232	87

VMA = voids in the mineral aggregate; VFB is the voids filled with the binder, TSR is the tensile strength ratio.

**Table 2:** A10E bituminous binder properties [36].

Property	Units	Test Method	Value
Softening Point	°C	AG:PT/T131	96
Torsional Recovery at 25 °C	%	AG:PT/T131	66
Viscosity at 165 °C	Pa·s	AG:PT/T131	0.693
Performance Grade	°C (at traffic level)	AASHTO TP 70	82 (E)
Elastic Recovery	% (82 °C, 3.2 kPa)	AASHTO TP 70	96

**Table 3:** Weathering chamber conditions.

Characteristic	Setting
Irradiation	50 w/m^2^
Irradiance Wavelength Range	300–400 nm
Chamber Temperature	70 °C
Humidity %	0%
Spray	Off.

**Table 4:** Iatroscan soaking and drying procedures.

Development Tank	Soaking Time	Drying Time/Temperature
Hexane	24 min	2 min at room temperature
Toluene	8.5 min	2 min at 110 °C 7 min at room temperature
Dichloromethane/methanol	2 min	2 min at 110 °C 5 min at room temperature

**Table 5:** Average complex shear modulus (Pa) at 1 Hz reference frequency.

Temperature (°C)	Oven			UV Chamber		
	Top	Middle	Ratio	Top	Middle	Ratio
30	5.69 × 10^5^	2.79 × 10^5^	2.0	1.96 × 10^6^	5.08 × 10^5^	3.9
35	2.75× 10^5^	1.32 × 10^5^	2.1	1.28 × 10^6^	3.23 × 10^5^	4.0
40	1.36 × 10^5^	6.62 × 10^4^	2.1	7.91 × 10^5^	1.66 × 10^5^	4.8
45	6.93× 10^4^	3.51 × 10^4^	2.0	4.72 × 10^5^	8.79 × 10^4^	5.4
50	3.62 × 10^4^	1.93 × 10^4^	1.9	2.78 × 10^5^	4.78 × 10^4^	5.8
55	1.90 × 10^4^	8.47 × 10^3^	2.2	1.47 × 10^5^	2.07 × 10^4^	7.1
60	1.08 × 10^4^	5.19 × 10^3^	2.1	8.50 × 10^4^	1.24 × 10^4^	6.9
65	6.33 × 10^3^	3.28 × 10^3^	1.9	5.05 × 10^4^	7.55 × 10^3^	6.7
70	3.83 × 10^3^	2.15 × 10^3^	1.8	3.10 × 10^4^	4.76 × 10^3^	6.5

**Table 6:** Iatroscan SARA analysis results of oven aged samples.

Sample/Position	% Saturates	% Aromatics	% Resins	% Asphaltenes
1/Top	6.2	32.5	40.9	20.4
1/Middle	6.2	45.1	27.3	21.4
1/Bottom	5.7	35.7	35.8	22.8
2/Top	6.1	29.8	45.9	18.2
2/Middle	6.6	46.8	22.3	24.3
2/Bottom	5.3	41.1	31.8	21.8
3/Top	6.4	29.1	46.7	17.8
3/Middle	5.7	31.1	41.8	21.4
3/Bottom	6.1	29.3	42.5	22.1

**Table 7:** Iatroscan SARA analysis results of UV aged samples.

Sample/Position	% Saturates	% Aromatics	% Resins	% Asphaltenes
1/Top	5.7	22.3	49.9	22.1
1/Middle	6.3	36.1	33.4	24.2
1/Bottom	5.9	29.1	39.9	25.1
2/Top	6.1	20.9	58.9	14.1
2/Middle	6.1	30.2	44.2	19.5
2/Bottom	6.1	21.7	53.1	19.1
3/Top	5.6	18.9	59.7	15.8
3/Middle	5.6	33.8	38.7	21.9
3/Bottom	5.4	27.5	45.8	21.3

**Table 8:** Aging Index from FTIR analysis.

Sample	AI_CO_	AI_SO_	AI_CH3_	AI(FTIR)
Oven Top	0.031	0.063	0.090	1.042
Oven Middle	0.025	0.065	0.089	1.018
Oven Bottom	0.038	0.064	0.092	1.004
UV Top	0.031	0.080	0.102	1.085
UV Middle	0.027	0.066	0.100	0.931
UV Bottom	0.034	0.067	0.108	0.943

## Data Availability

All data are presented within the manuscript. The original data are available from the corresponding author.
